# Supplementary material for: The Evolution and Characterization of the RNA Interference Pathways in Lophotrochozoa
Source: Genome Biol Evol. 2024 May 7;16(5):evae098. doi: 10.1093/gbe/evae098 (PMC11114477; doi:10.1093/gbe/evae098)
Supplement: evae098_Supplementary_Data [file evae098_supplementary_data.zip › Supplementary_materials.zip!/selected_miRNAs/NoGe_selected_miRNAs/mirdeep_pdf_files/NMRB01002279.1_Notospermus_geniculatus_strain_Ushimado_scaffold2279,_whole_genome_shotgun_sequence_37102.pdf]

Provisional ID	:	NMRB01002279.1_Notospermus_geniculatus_strain_Ushimado_scaffold2279,_whole_genome_shotgun_sequence_37102
Score total	:	62177.5
Score for star read(s)	:	3.9
Score for read counts	:	62169.2
Score for mfe	:	1.4
Score for randfold	:	0
Score for cons. seed	:	3
Total read count	:	121954
Mature read count	:	121926
Loop read count	:	0
Star read count	:	28

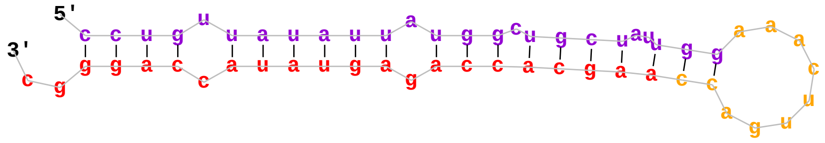

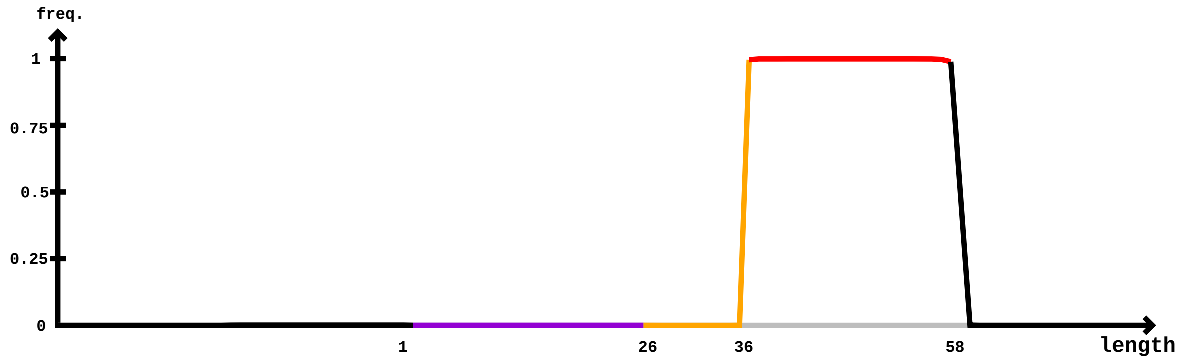

Star Mature

5'- auaccuggaaucaaguugugcauccuaucuaguguccuguuauauuauggcugcuauuggaaacuugaccaagcaccagaguauaccagggcagcacugggacacccacagc -3'	obs		
auaccuggaaucaaguugugcauccuaucuaguguccuguuauauuauggcugcuauuggaaacuugaccaagcaccagaguauaccagggcagcacugggacacccacagc	exp		
..............((((((..(((((.....(((((((.((((((.(((.((((..(((........)))))))))).)))))).)))))))....)))))....))))))	reads	mm	sample
................ugugcauccuaucuagug..	1	0	Mm2
................ugugcauccuaucuagugG...	3	1	Mm2
................ugugcauccuaucuagugu...	7	0	Mm2
.................gugcauccuaucuagugu...	1	0	Mm2
...................................ccuguuauauuauggcugcuauugg..	7	0	Mm2
...................................ccuguuauauuauggcugcuauugga...	3	0	Mm2
....................................cuguuauauuauggcugcuauugga...	1	0	Mm2
....gaUcaagcaccagaguauaccagggc....................	1	1	Mm2
....caagcaccagaguauaccagg......................	1	0	Mm2
....caagcacAagaguauaccagggc....................	1	1	Mm2
....caagcaccagaguauaccagggc....................	26	0	Mm2
....aagcaccagaguauaccag.......................	4	0	Mm2
....aagcaccagaguauaccagg......................	48	0	Mm2
....aagcaccagUguauaccaggg.....................	1	1	Mm2
....aagcaccagaguauacUaggg.....................	1	1	Mm2
....Uagcaccagaguauaccaggg.....................	1	1	Mm2
....aagcaccagaguauaccaggC.....................	6	1	Mm2
....aagcaccagaguauaccaUgg.....................	1	1	Mm2
....aagcaccagaguauaccCggg.....................	2	1	Mm2
....aagcaccagaguauacAaggg.....................	2	1	Mm2
....aagcaccagaguauaccaggg.....................	246	0	Mm2
....aagcaccagaguaGaccagggc....................	4	1	Mm2
....aagcUccagaguauaccagggc....................	12	1	Mm2
....aagcacNagaguauaccagggc....................	8	1	Mm2
....aagcacAagaguauaccagggc....................	1	1	Mm2
....aagcaccagaguauaccaggCc....................	3	1	Mm2
....aagcaccagaguauacGagggc....................	105	1	Mm2
....aagcaccagaguauaccaggUc....................	18	1	Mm2
....aagcaccagaAuauaccagggc....................	111	1	Mm2
....aagcaccagaguauacUagggc....................	237	1	Mm2
....aagcaccaAaguauaccagggc....................	9	1	Mm2
....aagcaccagagGauaccagggc....................	4	1	Mm2
....aUgcaccagaguauaccagggc....................	4	1	Mm2

```
                                            Star                                   Mature
auaccuggaaucaaguugugcauccuaucuaguguccuguuauauuauggcugcuauuggaaacuugaccaagcaccagaguauaccagggcagcacugggacacccacagc
......................................................................aagcaccagagCauaccagggc....................   7      1    Mm2
......................................................................aagcaccUgaguauaccagggc....................   5      1    Mm2
......................................................................aagcaccagaguauaGcagggc....................   1      1    Mm2
......................................................................aagcaccaguGuaccagggc....................   6      1    Mm2
......................................................................aagcaccagaguauaUcagggc....................   16     1    Mm2
......................................................................aagcaccagaUuauaccagggc....................   59     1    Mm2
......................................................................Uagcaccagaguauaccagggc....................   45     1    Mm2
......................................................................aagUaccagaguauaccagggc....................   8      1    Mm2
......................................................................aagcaUcagaguauaccagggc....................   10     1    Mm2
......................................................................aagcaccCgaguauaccagggc....................   1      1    Mm2
......................................................................aagcaccagaguauaccagggU....................   103    1    Mm2
......................................................................aagcacGagaguauaccagggc....................   6      1    Mm2
......................................................................aagcaccagaguauaccUgggc....................   222    1    Mm2
......................................................................aagcaccagaguauaAcagggc....................   3      1    Mm2
......................................................................aagcaccagaguauaccagggc....................   73220  0    Mm2
......................................................................aagcaccagaguauaccNgggc....................   1      1    Mm2
......................................................................aagcaccagaguauaccaAggc....................   7      1    Mm2
......................................................................aagcaccagaguauaccGgggc....................   219    1    Mm2
......................................................................Nagcaccagaguauaccagggc....................   28     1    Mm2
......................................................................aaCcaccagaguauaccagggc....................   8      1    Mm2
......................................................................aagcaccaguCuaccagggc....................   1      1    Mm2
......................................................................aagcacUagaguauaccagggc....................   19     1    Mm2
......................................................................aagcaccagaguauaccagggA....................   13     1    Mm2
......................................................................aagcaccagUguauaccagggc....................   15     1    Mm2
......................................................................aagcaccagaguauaccCgggc....................   206    1    Mm2
......................................................................aagcaccagaguauaccaCggc....................   5      1    Mm2
......................................................................aagcaccagagAauaccagggc....................   8      1    Mm2
......................................................................aagGaccagaguauaccagggc....................   2      1    Mm2
......................................................................Gagcaccagaguauaccagggc....................   9      1    Mm2
......................................................................aagcaNcagaguauaccagggc....................   9      1    Mm2
......................................................................aagcaccagaguauaccaggAc....................   12     1    Mm2
......................................................................aagcaccaguUuaccagggc....................   3      1    Mm2
......................................................................aCgcaccagaguauaccagggc....................   3      1    Mm2
......................................................................aGgcaccagaguauaccagggc....................   7      1    Mm2
......................................................................aagcaccagaguauacAagggc....................   327    1    Mm2
......................................................................aagcaccaUaguauaccagggc....................   43     1    Mm2
......................................................................aagcaccagaguauaccagAgc....................   7      1    Mm2
......................................................................aaNcaccagaguauaccagggc....................   1      1    Mm2
......................................................................aagcaccagaguauGccagggc....................   3      1    Mm2
......................................................................aagcaccagaguauaccagggG....................   14     1    Mm2
......................................................................aagcGccagaguauaccagggc....................   10     1    Mm2
......................................................................aagcaAcagaguauaccagggc....................   4      1    Mm2
......................................................................aaAcaccagaguauaccagggc....................   25     1    Mm2
......................................................................aagcaccaguaCaccagggc....................   10     1    Mm2
......................................................................aagAaccagaguauaccagggc....................   12     1    Mm2
......................................................................aagcaccaguaAaccagggc....................   13     1    Mm2
......................................................................aaUcaccagaguauaccagggc....................   25     1    Mm2
......................................................................aagcaccagaguauaccaUggc....................   8      1    Mm2
......................................................................Cagcaccagaguauaccagggc....................   1      1    Mm2
......................................................................aagcaccagaCuauaccagggc....................   79     1    Mm2
......................................................................aagcaccagaguauaccagUgc....................   14     1    Mm2
......................................................................aagcaccagaguauaccagCgc....................   3      1    Mm2
......................................................................aagcaccagCguauaccagggc....................   4      1    Mm2
......................................................................aagcaccagaguauUccagggc....................   8      1    Mm2
......................................................................aagcaccagGguauaccagggc....................   15     1    Mm2
......................................................................aagcaccGgaguauaccagggc....................   13     1    Mm2
......................................................................aagcCccagaguauaccagggc....................   7      1    Mm2
......................................................................aagcaGcagaguauaccagggc....................   3      1    Mm2
......................................................................aagcaccagaguauaccagCgca...................   1      1    Mm2
......................................................................aagcaccagaCuauaccagggca...................   1      1    Mm2
......................................................................aagcaccagaguauaccagggcU...................   330    1    Mm2
......................................................................aagcaccagaguauacAagggca...................   2      1    Mm2
......................................................................aagcaccagaAuauaccagggca...................   1      1    Mm2
......................................................................aagcaccagaguauaccagggca...................   124    0    Mm2
......................................................................aagcaccagaguauaccagggcC...................   8      1    Mm2
......................................................................aagcaccagaguauaccGgggca...................   3      1    Mm2
......................................................................aagcaccagaguauaccagggcG...................   9      1    Mm2
......................................................................aagcaccagaguauaccUgggca...................   1      1    Mm2
......................................................................aagcaccagaguauaccagUgca...................   1      1    Mm2
......................................................................aagcaccagaguauaccaggUca...................   1      1    Mm2
```

Star Mature

auaccuggaaucaaguugugcauccuaucuagugucc uguuauauuauggcugcuauuggaaacuugaccaagcaccagaguauaccagggcagcacugggacacccacagc			
....aagcaccagaguauaccagggcaU..................	20	1	Mm2
....aagcaccagaguauaccagggcaA..................	6	1	Mm2
....aagcaccagaguauaccagggcUg..................	6	1	Mm2
....aagcaccagaguauaccagggcCg..................	1	1	Mm2
....agcaccagaguauaccaAggc....................	1	1	Mm2
....agcaccagaguauaccaCggc....................	1	1	Mm2
....agcaccagCguauaccagggc....................	2	1	Mm2
....agcaccagaguauacUagggc....................	1	1	Mm2
....agcaccaAaguauaccagggc....................	1	1	Mm2
....agcaccagagAauaccagggc....................	2	1	Mm2
....agcaccagaguauaccagggc....................	217	0	Mm2
....agcaccagaguauaccCgggc....................	3	1	Mm2
....agcaccagaguauaAcagggc....................	1	1	Mm2
....agcaccagaguauaccagggca...................	1	0	Mm2
....gcaccagaguauaccagggc....................	1	0	Mm2
....accagaguauaccagggc....................	3	0	Mm2
................ugugcauccuaucuagAgu...	1	1	Mm1
................ugugcauccuaucuagugu...	13	0	Mm1
...................................ccuguuauauuauggcugcuauugg..	9	0	Mm1
....aagcaccagaguauaccagg......................	33	0	Mm1
....aagcaccagaAuauaccagg......................	1	1	Mm1
....aagcaccagaguauaccGggg.....................	2	1	Mm1
....aagcaccagaguauaccaggg.....................	252	0	Mm1
....aagcaccagaguauacGaggg.....................	3	1	Mm1
....aagcaccagaguauaccCggg.....................	1	1	Mm1
....aagcaccagaguauacUaggg.....................	1	1	Mm1
....aagcaccagaguauCccaggg.....................	1	1	Mm1
....aagcaccagaguauaccUggg.....................	1	1	Mm1
....aagcaccGgaguauaccagggc....................	1	1	Mm1
....aagcaccagaguauaccGgggc....................	12	1	Mm1
....aagAaccagaguauaccagggc....................	1	1	Mm1
....aaCcaccagaguauaccagggc....................	1	1	Mm1
....aagcaccagaguauaccaUggc....................	1	1	Mm1
....aagcaccagaguauacGagggc....................	7	1	Mm1
....aagcaccagaguauaccagAgc....................	1	1	Mm1
....aaUcaccagaguauaccagggc....................	2	1	Mm1
....aagcaccagaguauacUagggc....................	14	1	Mm1
....aagcGccagaguauaccagggc....................	1	1	Mm1
....aagcaccagagCauaccagggc....................	1	1	Mm1
....aagcaccagaUuauaccagggc....................	5	1	Mm1
....aagUaccagaguauaccagggc....................	1	1	Mm1
....aGgcaccagaguauaccagggc....................	1	1	Mm1
....aagcaccagaguauaccagggA....................	8	1	Mm1
....aagcaccagaAuauaccagggc....................	7	1	Mm1
....aagcaccagGguauaccagggc....................	4	1	Mm1
....aagcaccagaguauaccCgggc....................	7	1	Mm1
....aagcaccagaguauaccagggc....................	3118	0	Mm1
....aagcaccagaguauacAagggc....................	10	1	Mm1
....aagcaccagaguauaccagggU....................	25	1	Mm1
....aagcaccaUaguauaccagggc....................	5	1	Mm1
....aagcacAagaguauaccagggc....................	2	1	Mm1
....aagcaccaAaguauaccagggc....................	1	1	Mm1
....aagcaccagaCuauaccagggc....................	1	1	Mm1
....Uagcaccagaguauaccagggc....................	4	1	Mm1
....aagcaccagaguauaccUgggc....................	10	1	Mm1
....aagcacUagaguauaccagggc....................	1	1	Mm1
....Nagcaccagaguauaccagggc....................	2	1	Mm1
....aagcaccagaguauaccagggcU...................	18	1	Mm1
....aagcaccagaguauacUagggca...................	1	1	Mm1
....aagcaccagaguauaccagggAa...................	8	1	Mm1
....aagcaccagaguauaccagggca...................	74	0	Mm1
....aagcaccagaguauaccagggcaA..................	2	1	Mm1
................ugugcauccuaucuagugG...	2	1	Fm1
................ugugcauccuaucuagCgu...	1	1	Fm1
................ugugcauccuaucuagugu...	12	0	Fm1
................ugGgcauccuaucuagugu...	1	1	Fm1
.................gugcauccuaucuagugu...	5	0	Fm1
...................................ccuguuauauuauggcug...	2	0	Fm1

Star Mature

```
auaccuggaaucaaguugugcauccuaucuaguguccuguuauauuauggcugcuauuggaaacuugaccaagcaccagaguauaccagggcagcacugggacacccacagc
...................................ccuguuauauuauggcugcuauugg....................................................   2      0   Fm1
.....................................................................caagcaccagaguauaccagggc...................   1      0   Fm1
......................................................................aagcaccagaguauaccagg......................   15     0   Fm1
......................................................................aagcaccagaguauaccGggg.....................   1      1   Fm1
......................................................................aagcaccagaguauaccaggU.....................   2      1   Fm1
......................................................................aagcaccagaguauacAaggg.....................   1      1   Fm1
......................................................................aagcaccagaguauaccaggg.....................   126    0   Fm1
......................................................................aagcaccagaguauaccUggg.....................   1      1   Fm1
......................................................................aagcaccagaguGuaccagggc....................   2      1   Fm1
......................................................................aagcaccagaguauaccaUggc....................   5      1   Fm1
......................................................................aagcaccagaguaCaccagggc....................   1      1   Fm1
......................................................................aagcaccagaguauaccGgggc....................   59     1   Fm1
......................................................................aagAaccagaguauaccagggc....................   4      1   Fm1
......................................................................aaAcaccagaguauaccagggc....................   5      1   Fm1
......................................................................Cagcaccagaguauaccagggc....................   1      1   Fm1
......................................................................aagcaccaUaguauaccagggc....................   11     1   Fm1
......................................................................aagcacNagaguauaccagggc....................   1      1   Fm1
......................................................................aCgcaccagaguauaccagggc....................   3      1   Fm1
......................................................................aagcaccagaguauaccaggCc....................   2      1   Fm1
......................................................................aagcGccagaguauaccagggc....................   1      1   Fm1
......................................................................aagcUccagaguauaccagggc....................   3      1   Fm1
......................................................................aagcaccagaguauUccagggc....................   1      1   Fm1
......................................................................aagcaccagaUuauaccagggc....................   7      1   Fm1
......................................................................aagcaccagaguauacUagggc....................   38     1   Fm1
......................................................................aagcaccagaguauaccagAgc....................   2      1   Fm1
......................................................................aagcaccagGguauaccagggc....................   5      1   Fm1
......................................................................aagcaccagUguauaccagggc....................   6      1   Fm1
......................................................................aagcaccagaguauaUcagggc....................   12     1   Fm1
......................................................................aagcaccGgaguauaccagggc....................   5      1   Fm1
......................................................................aagcaccagaguauaccagggA....................   4      1   Fm1
......................................................................aagcaccagaguauacGagggc....................   11     1   Fm1
......................................................................aagcaccagaguauacAagggc....................   67     1   Fm1
......................................................................aagcaccagaguauaccagggU....................   28     1   Fm1
......................................................................aUgcaccagaguauaccagggc....................   1      1   Fm1
......................................................................aagcaccagaguauaccagCgc....................   1      1   Fm1
......................................................................aagcCccagaguauaccagggc....................   1      1   Fm1
......................................................................aagcaccagCguauaccagggc....................   2      1   Fm1
......................................................................aagcaccagaguauaccCgggc....................   46     1   Fm1
......................................................................aagcaccagaguauaccUgggc....................   23     1   Fm1
......................................................................aaCcaccagaguauaccagggc....................   3      1   Fm1
......................................................................aagcacAagaguauaccagggc....................   2      1   Fm1
......................................................................aagUaccagaguauaccagggc....................   4      1   Fm1
......................................................................aagcaccagaguaAaccagggc....................   1      1   Fm1
......................................................................aagcacUagaguauaccagggc....................   2      1   Fm1
......................................................................aagGaccagaguauaccagggc....................   1      1   Fm1
......................................................................Uagcaccagaguauaccagggc....................   10     1   Fm1
......................................................................aagcaccagaguauaccaggUc....................   6      1   Fm1
......................................................................aagcaccagaguauaccagUgc....................   2      1   Fm1
......................................................................aGgcaccagaguauaccagggc....................   5      1   Fm1
......................................................................aagcaccagaCuauaccagggc....................   11     1   Fm1
......................................................................aagcaccCgaguauaccagggc....................   1      1   Fm1
......................................................................aagcaccagaguaGaccagggc....................   2      1   Fm1
......................................................................aagcaNcagaguauaccagggc....................   2      1   Fm1
......................................................................Gagcaccagaguauaccagggc....................   4      1   Fm1
......................................................................aagcaccagaguauaccagggG....................   3      1   Fm1
......................................................................aagcaccagagAauaccagggc....................   2      1   Fm1
......................................................................aagcaccagaguauaccagggc....................   15552  0   Fm1
......................................................................Nagcaccagaguauaccagggc....................   11     1   Fm1
......................................................................aaUcaccagaguauaccagggc....................   2      1   Fm1
......................................................................aagcaccagagCauaccagggc....................   1      1   Fm1
......................................................................aagcaccagaAuauaccagggc....................   30     1   Fm1
......................................................................aagcaccagaguauaccagggca...................   93     0   Fm1
......................................................................aagcaccagaguauaccagggAa...................   4      1   Fm1
......................................................................aagcaccagaguauaccagggcU...................   237    1   Fm1
......................................................................aagcaccagaguauaccagggcG...................   16     1   Fm1
......................................................................aagcaccagaguauaccUgggca...................   1      1   Fm1
......................................................................aagcaccagaguauaccCgggca...................   1      1   Fm1
......................................................................aagcaccagaguauaccagggcC...................   1      1   Fm1
......................................................................aagcaccagaguauaccagggcaA..................   1      1   Fm1
......................................................................aagcaccagaguauaccagggcaU..................   29     1   Fm1
```

Star Mature

```
auaccuggaaucaaguugugcauccuaucuaguguccuguuauauuauggcugcuauuggaaacuugaccaagcaccagaguauaccagggcagcacugggacacccacagc
.......................................................................agcaccagaguauaccagggc....................   18    0   Fm1
.......................................................................agcaccagagCauaccagggc....................   1     1   Fm1
.......................................................................agcaccagagAauaccagggc....................   1     1   Fm1

.....................................................................caagcaccagaguauaccagggc....................   4     0   Fm2
.....................................................................caagcaccagaguauaccCgggc....................   1     1   Fm2
......................................................................aagcaccagaguauaccagg......................   4     0   Fm2
......................................................................aagcaccagaguauacUaggg.....................   1     1   Fm2
......................................................................aagcaccagaguauaccaggg.....................   32    0   Fm2
......................................................................aagcaccagaguauaccaggU.....................   6     1   Fm2
......................................................................aagcaccagaguauaccagUg.....................   1     1   Fm2
......................................................................aagcaccagaguauaccUggg.....................   1     1   Fm2
......................................................................aagcaccagaguauacUagggc....................   20    1   Fm2
......................................................................aagcaAcagaguauaccagggc....................   1     1   Fm2
......................................................................aagcUccagaguauaccagggc....................   1     1   Fm2
......................................................................Gagcaccagaguauaccagggc....................   3     1   Fm2
......................................................................Uagcaccagaguauaccagggc....................   2     1   Fm2
......................................................................aagcaccagGguauaccagggc....................   2     1   Fm2
......................................................................aagcaccagaguauaccaAggc....................   1     1   Fm2
......................................................................aagcacGagaguauaccagggc....................   1     1   Fm2
......................................................................aagcaccagaguauacAagggc....................   28    1   Fm2
......................................................................aagcaccagaguauaccaCggc....................   1     1   Fm2
......................................................................aagcaccagaguauaccagAgc....................   1     1   Fm2
......................................................................aagcaccUgaguauaccagggc....................   2     1   Fm2
......................................................................aagcaccagaguauaGcagggc....................   1     1   Fm2
......................................................................aagcaccagaguauaccagggA....................   6     1   Fm2
......................................................................aagcaccagaguauacGagggc....................   9     1   Fm2
......................................................................Nagcaccagaguauaccagggc....................   1     1   Fm2
......................................................................aagcaNcagaguauaccagggc....................   2     1   Fm2
......................................................................aagcaccagaguauaccagUgc....................   3     1   Fm2
......................................................................aagcacUagaguauaccagggc....................   1     1   Fm2
......................................................................aagcaccaUaguauaccagggc....................   14    1   Fm2
......................................................................aagcaccagaguauaAcagggc....................   1     1   Fm2
......................................................................aagcaccagaguauaccaUggc....................   1     1   Fm2
......................................................................aagcaUcagaguauaccagggc....................   2     1   Fm2
......................................................................aagcCccagaguauaccagggc....................   1     1   Fm2
......................................................................aagcaccGgaguauaccagggc....................   1     1   Fm2
......................................................................aagcaccagaUuauaccagggc....................   6     1   Fm2
......................................................................aagcaccagaguauaccUgggc....................   22    1   Fm2
......................................................................aagcaccagaguauaccagggU....................   14    1   Fm2
......................................................................aagcaccagaguauaccGgggc....................   24    1   Fm2
......................................................................aagcaccagaguauaccagCgc....................   1     1   Fm2
......................................................................aagcaccagaguauacNagggc....................   1     1   Fm2
......................................................................aUgcaccagaguauaccagggc....................   3     1   Fm2
......................................................................aagcGccagaguauaccagggc....................   1     1   Fm2
......................................................................aagcaccagaAuauaccagggc....................   12    1   Fm2
......................................................................aagcaccaAaguauaccagggc....................   1     1   Fm2
......................................................................aagcaccagaguauaccagggc....................   6204  0   Fm2
......................................................................aagcaccagaguauaccaggAc....................   2     1   Fm2
......................................................................aagcaccagaguauaccagggG....................   2     1   Fm2
......................................................................aagcaccagaguauaUcagggc....................   1     1   Fm2
......................................................................aagcaccagaCuauaccagggc....................   5     1   Fm2
......................................................................aagcaccagaguaCaccagggc....................   8     1   Fm2
......................................................................aagcacAagaguauaccagggc....................   1     1   Fm2
......................................................................aaAcaccagaguauaccagggc....................   2     1   Fm2
......................................................................aaUcaccagaguauaccagggc....................   1     1   Fm2
......................................................................aagcaccagaguauaccCgggc....................   17    1   Fm2
......................................................................aagcaccagaguauacUagggca...................   1     1   Fm2
......................................................................aagcaccagaguauaccagggcU...................   24    1   Fm2
......................................................................aagcaccagaguauaccagggca...................   61    0   Fm2
......................................................................aagcaccagaguauaccagggcaU..................   11    1   Fm2
.......................................................................agcaccagagCauaccagggc....................   1     1   Fm2
.......................................................................agcaccagaguauaccagggc....................   15    0   Fm2

................ugugcauccuaucuagugu.............................................................................   4     0   Mm3
.................gugcauccuaucuagugu.............................................................................   2     0   Mm3
...................................ccuguuauauuauggcugcuauugg....................................................   2     0   Mm3
...................................ccuguuauauuauggcugcuauugga...................................................   1     0   Mm3
....................................cuguuauauuauggcugcuauugg....................................................   1     0   Mm3
.....................................................................caagcaccagaguauaccaggU.....................   1     1   Mm3
```

```
                                    Star                                  Mature
auaccuggaaucaaguugugcauccuaucuagugucuguuauauuauggcugcuauuggaaacuugaccaagcaccagaguauaccagggcagcacugggacacccacagc
.....................................................................caagcaccagaguauaccaggg.....................   1      0    Mm3
.....................................................................caagcaccagaguauaccagggc....................   2      0    Mm3
.....................................................................caagcaccagaguauaccagggcag..................   1      0    Mm3
......................................................................aagcaccagaguauaccag.......................   1      0    Mm3
......................................................................aagcaccagaguauaccagg......................   18     0    Mm3
......................................................................aagcaccagaguauaccaggg.....................   128    0    Mm3
......................................................................aagcaccagaguauaccaggC.....................   1      1    Mm3
......................................................................aagcaccagaguauaccaggA.....................   1      1    Mm3
......................................................................aagcaccagaguauaccaggU.....................   3      1    Mm3
......................................................................aagcaccagaCuauaccaggg.....................   1      1    Mm3
......................................................................aagAaccagaguauaccaggg.....................   1      1    Mm3
......................................................................aagcaccagaguauaccaggCc....................   2      1    Mm3
......................................................................aagcacNagaguauaccagggc....................   3      1    Mm3
......................................................................aagcaccagaguauacUagggc....................   21     1    Mm3
......................................................................aagcaccagaguauaccagggU....................   17     1    Mm3
......................................................................aagcaccagaguauaUcagggc....................   1      1    Mm3
......................................................................aagcaccagaguauaccaggUc....................   5      1    Mm3
......................................................................aagcaccagaCuauaccagggc....................   6      1    Mm3
......................................................................aagcaccagCguauaccagggc....................   1      1    Mm3
......................................................................aaCcaccagaguauaccagggc....................   1      1    Mm3
......................................................................aagcaccagaguauaccUgggc....................   24     1    Mm3
......................................................................aagcaccagaguauaccagggc....................   8173   0    Mm3
......................................................................aagcaccUgaguauaccagggc....................   2      1    Mm3
......................................................................aGgcaccagaguauaccagggc....................   1      1    Mm3
......................................................................Uagcaccagaguauaccagggc....................   8      1    Mm3
......................................................................aagcaccagaUuauaccagggc....................   7      1    Mm3
......................................................................aagcaccCgaguauaccagggc....................   1      1    Mm3
......................................................................aagcacUagaguauaccagggc....................   2      1    Mm3
......................................................................Nagcaccagaguauaccagggc....................   5      1    Mm3
......................................................................aagcaccagaguauGccagggc....................   1      1    Mm3
......................................................................Gagcaccagaguauaccagggc....................   1      1    Mm3
......................................................................aagcaccagaguauaccCgggc....................   26     1    Mm3
......................................................................aagcaccagaguauaGcagggc....................   1      1    Mm3
......................................................................aagcaccagaguauacGagggc....................   15     1    Mm3
......................................................................aagcaccagaguauUccagggc....................   2      1    Mm3
......................................................................aagcaccagaguauaccagggG....................   1      1    Mm3
......................................................................aagcaccagaguGuaccagggc....................   1      1    Mm3
......................................................................aagcaccagaguauaccaggAc....................   1      1    Mm3
......................................................................aaUcaccagaguauaccagggc....................   3      1    Mm3
......................................................................Cagcaccagaguauaccagggc....................   1      1    Mm3
......................................................................aagcaccagaguaAaccagggc....................   1      1    Mm3
......................................................................aagcaccagaguauaccGgggc....................   26     1    Mm3
......................................................................aagcaccagaguauaccagAgc....................   2      1    Mm3
......................................................................aagcaccaUaguauaccagggc....................   16     1    Mm3
......................................................................aagcaccagaguaCaccagggc....................   1      1    Mm3
......................................................................aCgcaccagaguauaccagggc....................   1      1    Mm3
......................................................................aagcaccagaguauaccagggA....................   2      1    Mm3
......................................................................aagcaccagaguauacAagggc....................   27     1    Mm3
......................................................................aagcaccagaguauaccagUgc....................   2      1    Mm3
......................................................................aaAcaccagaguauaccagggc....................   1      1    Mm3
......................................................................aagcaUcagaguauaccagggc....................   2      1    Mm3
......................................................................aagcacGagaguauaccagggc....................   1      1    Mm3
......................................................................aagcaccagaAuauaccagggc....................   16     1    Mm3
......................................................................aagcaccagaguauaccaUggc....................   1      1    Mm3
......................................................................aagcaccagUguauaccagggc....................   2      1    Mm3
......................................................................aagcaccagaguaGaccagggc....................   3      1    Mm3
......................................................................aUgcaccagaguauaccagggc....................   3      1    Mm3
......................................................................aagcaccagaguauaccagCgca...................   1      1    Mm3
......................................................................aagcaccagaguauaccagggAa...................   1      1    Mm3
......................................................................aagcaccagaguauaccagggcG...................   2      1    Mm3
......................................................................aagcaccagaguauaccagggcC...................   1      1    Mm3
......................................................................aagcaccagaguauaccagggcU...................   96     1    Mm3
......................................................................aagcaccagaguauaccagggca...................   25     0    Mm3
......................................................................aagcaccaCaguauaccagggcag..................   1      1    Mm3
......................................................................aagcaccagaguauaccagggcaU..................   4      1    Mm3
......................................................................aagcaccagaguauaccagggcUg..................   5      1    Mm3
......................................................................aagcaccagaguauaccagggcag..................   1      0    Mm3
......................................................................aagcaccagaguauaccagggcaA..................   3      1    Mm3
......................................................................aagcaccagaguauaccagggcagU.................   1      1    Mm3
.......................................................................agcaccagagCauaccagggc....................   1      1    Mm3
```

```
                                    Star                              Mature
auaccuggaaucaaguugugcauccuaucuaguguccuguuauauuauggcugcuauuggaaacuugaccaagcaccagaguauaccagggcagcacugggacacccacagc
.......................................................................agcaccagaguauaccagggc....................  23    0    Mm3
........................................................................gcaccagaguauaccaggg.....................  1     0    Mm3
........................................................................gcaccagaguauaccagggc....................  3     0    Mm3
.........................................................................caccagaguauaccagggca...................  1     0    Mm3
..........................................................................accagaguauaccagggc....................  2     0    Mm3

................ugugcauccuaucuagugu.............................................................................  40    0    Fm3
................ugugcauccuaucuagAgu.............................................................................  1     1    Fm3
................ugugcauccuaucuaguCu.............................................................................  1     1    Fm3
.................gugcauccuaucuagugu.............................................................................  16    0    Fm3
......................................................................aagcaccagaguauacca........................  3     0    Fm3
......................................................................aagcaccagaguauaccag.......................  13    0    Fm3
......................................................................aagcaccagaguauaccUg.......................  1     1    Fm3
......................................................................Uagcaccagaguauaccagg......................  1     1    Fm3
......................................................................aagcaccagaAuauaccagg......................  1     1    Fm3
......................................................................aagcaccagaguauaccagg......................  75    0    Fm3
......................................................................aagcaccagaguauaccaggg.....................  225   0    Fm3
......................................................................aagcaccagaguauaccUggg.....................  1     1    Fm3
......................................................................aagcaccagaguauaAcaggg.....................  1     1    Fm3
......................................................................aagcaccagaguauacUaggg.....................  1     1    Fm3
......................................................................aagcaccagaAuauaccaggg.....................  1     1    Fm3
......................................................................aagcaccagaguauacAaggg.....................  1     1    Fm3
......................................................................aagcaccagaguauaccUgggc....................  31    1    Fm3
......................................................................aagcaccagagCauaccagggc....................  5     1    Fm3
......................................................................aagcaccagaguaGaccagggc....................  1     1    Fm3
......................................................................aagcaccagaguauaccagggA....................  14    1    Fm3
......................................................................aagcacAagaguauaccagggc....................  1     1    Fm3
......................................................................Nagcaccagaguauaccagggc....................  4     1    Fm3
......................................................................aagcaccagaCuauaccagggc....................  7     1    Fm3
......................................................................Gagcaccagaguauaccagggc....................  2     1    Fm3
......................................................................aagcaccagaguauacAagggc....................  43    1    Fm3
......................................................................aagcaUcagaguauaccagggc....................  2     1    Fm3
......................................................................aagcaccagaguGuaccagggc....................  8     1    Fm3
......................................................................aagcaccagaguauaccaCggc....................  1     1    Fm3
......................................................................aagcaccagaguauaccaAggc....................  2     1    Fm3
......................................................................aagcaccagaguauacUagggc....................  34    1    Fm3
......................................................................aagcaccagaguaAaccagggc....................  1     1    Fm3
......................................................................aagcaccagaguauaccagUgc....................  4     1    Fm3
......................................................................Uagcaccagaguauaccagggc....................  7     1    Fm3
......................................................................aagcaccagGguauaccagggc....................  2     1    Fm3
......................................................................aagcacGagaguauaccagggc....................  2     1    Fm3
......................................................................aagcaccagaguauaccaUggc....................  4     1    Fm3
......................................................................aagcaccagaguauaAcagggc....................  4     1    Fm3
......................................................................aagcaccagaguauaccaggCc....................  1     1    Fm3
......................................................................aagcaccagaguUuaccagggc....................  1     1    Fm3
......................................................................aagcGccagaguauaccagggc....................  1     1    Fm3
......................................................................aagcaccagaguauaUcagggc....................  8     1    Fm3
......................................................................aagcaccagaguauaccagggc....................  8949  0    Fm3
......................................................................aaUcaccagaguauaccagggc....................  2     1    Fm3
......................................................................aagcaccagaguauaccagggU....................  22    1    Fm3
......................................................................Cagcaccagaguauaccagggc....................  1     1    Fm3
......................................................................aagcaccUgaguauaccagggc....................  1     1    Fm3
......................................................................aagcaccagaguauaccGgggc....................  33    1    Fm3
......................................................................aagcaccagUguauaccagggc....................  3     1    Fm3
......................................................................aagcaccagaUuauaccagggc....................  8     1    Fm3
......................................................................aagcaccGgaguauaccagggc....................  2     1    Fm3
......................................................................aaAcaccagaguauaccagggc....................  5     1    Fm3
......................................................................aagcaccagaguauaccaggAc....................  3     1    Fm3
......................................................................aagcaccaUaguauaccagggc....................  33    1    Fm3
......................................................................aagcaccagaguauaccaggUc....................  2     1    Fm3
......................................................................aagcaccagaguauacGagggc....................  13    1    Fm3
......................................................................aagcacNagaguauaccagggc....................  2     1    Fm3
......................................................................aUgcaccagaguauaccagggc....................  2     1    Fm3
......................................................................aagcaccagaguauaccCgggc....................  25    1    Fm3
......................................................................aagcUccagaguauaccagggc....................  2     1    Fm3
......................................................................aagcaccagagAauaccagggc....................  1     1    Fm3
......................................................................aagAaccagaguauaccagggc....................  1     1    Fm3
......................................................................aagcaccagaAuauaccagggc....................  12    1    Fm3
......................................................................aagcaNcagaguauaccagggc....................  1     1    Fm3
......................................................................aCgcaccagaguauaccagggc....................  2     1    Fm3
```

Star **Mature**

```
auaccuggaaucaaguugugcauccuaucuaguguccuguuauauuauggcugcuauuggaaacuugaccaagcaccagaguauaccagggcagcacugggacacccacagc
......................................................................aagcaccaAaguauaccagggc....................   3    1    Fm3
......................................................................aagcaccagaguauaccagggAa...................   20   1    Fm3
......................................................................aagcaccagaguauaccagggcU...................   32   1    Fm3
......................................................................aagcaccaUaguauaccagggca...................   8    1    Fm3
......................................................................aagcaccagaguauaccagggca...................   35   0    Fm3
......................................................................aagcaccagaguauaccagggcaU..................   6    1    Fm3
......................................................................aagcaccagaguauaccagggcaA..................   1    1    Fm3
.......................................................................agcaccagagCauaccagggc....................   1    1    Fm3
.......................................................................aAcaccagaguauaccagggc....................   1    1    Fm3
.......................................................................agcaccagaguauaccagggc....................   52   0    Fm3
..........................................................................accagaguauaccagggc....................   2    0    Fm3
```
